# Randomized Trials to Optimize Treatment of Multidrug-Resistant Tuberculosis: The time for action is now

**DOI:** 10.1371/journal.pmed.0040292

**Published:** 2007-11-06

**Authors:** Carole D Mitnick, Kenneth G Castro, Mark Harrington, Leonard V Sacks, William Burman

## Abstract

The time is now right for randomized trials of MDR-TB, say the authors, as the expansion of MDR-TB programs provides the setting in which trials can be implemented.

## The Pressing Need for MDR-TB Clinical Trials

Drug-resistant strains of Mycobacterium tuberculosis may account for 10% of the 8 million new cases of tuberculosis (TB) that occur annually. Systematic surveys have been undertaken in at least 90 countries. Drug-resistant isolates were found in every site, and multidrug-resistant tuberculosis (MDR-TB; resistant to at least isoniazid and rifampin) in all but eight [1]. Extensively drug-resistant tuberculosis (XDR-TB)—disease caused by MDR strains that are also resistant to at least one fluoroquinolone and one or more injectable agents [2]—has been reported in at least 37 countries, with very poor treatment outcomes [3–5]. Increasing concern about resistance has redoubled interest in strategies to control drug-resistant TB, especially in settings of high HIV prevalence [6].

There is, therefore, increased urgency for clinical trials that will identify safe and effective regimens for patients who have no treatment options. Furthermore, although the feasibility and cost-effectiveness of treating patients with MDR-TB in resource-constrained countries is well established [7–13], outcomes of MDR-TB treatment remain suboptimal. MDR-TB can be lethal; 5%–20% of HIV-uninfected patients [7,9–11,14] and 66% of HIV-infected patients die during treatment [15]. MDR-TB treatment lasts between 18 and 24 months, and adverse events are common [16]. As a result, the combined frequency of cure and completion often remains below 50% [7,17,18]. Even when therapy is designed with access to the full complement of antituberculosis agents presently available, outcomes rarely approach the target for TB treatment success (cure among at least 85% of patients initiating therapy) [14,19]. The long duration and toxicity of current MDR-TB regimens will impede achievement of the goal of treating nearly 1.6 million MDR-TB patients by 2015, set out in the Global Plan to Stop TB [20]. In addition, the poor outcomes of current regimens mean that many of those treated will develop chronic, highly resistant forms of TB that have a high mortality rate and can be transmitted to others.

**Summary Points**

The time is now right for randomized trials of MDR-TB:

- The burden of multidrug-resistant tuberculosis (MDR-TB) is growing, and the standard-of-care treatment is long, toxic, and frequently unsuccessful.
- The need for additional research is clearly illustrated by suboptimal treatment outcomes and limited estimates of the burden of MDR-TB and extensively drug-resistant TB.
- The expansion of MDR-TB treatment programs provides the settings in which trials can be implemented.
- For the first time in 30 years, several new drug classes that hold promise for MDR-TB treatment are under development; these new agents should be evaluated in parallel for drug-resistant and drug-susceptible TB.
- Concerns about MDR-TB can help mobilize additional funding for TB drug development, while clinical trials for MDR-TB will likely allow accelerated regulatory approval for new agents.

## The Past

The history of the development of current, short-course standardized treatment for drug-susceptible TB is instructive for developing regimens to treat MDR-TB. Identified through a series of randomized clinical trials, the short-course approach offered dramatic benefits over earlier standards of care—treatment duration decreased from 24 to six months while outcomes improved [21]. In contrast, guidelines for management of MDR-TB [22] are based on expert opinion and results of cohort and case series analyses, since no large-scale randomized trials of treatment have been conducted.

As in the case of drug-susceptible TB, randomized controlled trials would provide a strong evidence base for developing treatment regimens for MDR-TB using specific drug combinations and durations of therapy (of injectable and entire regimen). The global expansion of treatment for drug-resistant disease would thus be improved. Box 1 provides an example of the consequences of not subjecting to controlled clinical trials a new class of agents with documented antituberculosis activity.

**Box 1: Fluoroquinolones: A Highly Active Drug Class with No Evidence on Optimization of Use**

As new drugs with antituberculosis activity have been identified, they have been incorporated into MDR-TB treatment regimens. The use of fluoroquinolones for MDR-TB treatment exemplifies the problems of changing therapy in the absence of evidence from rigorously conducted clinical trials. The fluoroquinolones are considered to be the most important drug class in the treatment of MDR-TB, based on their in vitro activity and favorable results in case series.

However, there is agreement neither on the best fluoroquinolone for TB treatment nor on the optimal duration of fluoroquinolone-containing MDR-TB treatment regimens. The current recommended duration of treatment for MDR-TB (18–24 months) derives from the pre-fluoroquinolone era. In light of the excellent activity of the fluoroquinolones, it is conceivable that their inclusion could shorten treatment for MDR-TB; inclusion of class members with greater antituberculosis activity could shorten treatment further.

In the absence of data from controlled trials and in the face of such high stakes, however, clinicians are understandably reluctant to shorten fluoroquinolone-containing MDR-TB regimens. Without evidence from clinical trials to show the benefits of more active—and more expensive—fluoroquinolones in MDR-TB regimens, cost considerations alone have precluded the use of these newer agents.

Without clinical trials, new agents with demonstrable activity will be used for MDR-TB treatment. Persistent questions about their efficacy, dosing, and toxicity, however, will lead to even greater heterogeneity in treatment practices—hardly the way to expand evidence-based MDR-TB treatment programs around the globe.

Randomized trials of MDR-TB treatment have been long considered impossible for three reasons. First, the political will to improve treatment for MDR-TB has been lacking, in part due to perceptions that the disease has limited epidemiologic importance (i.e., few cases and poor fitness of resistant organisms). Second, patients with MDR-TB show great variability in the extent of disease and degree of known intolerance to antituberculosis agents; their infecting isolates, moreover, vary in the degree of drug resistance. These differences were thought to preclude the implementation of classical clinical trials, which compare the efficacy of fixed regimens. Finally, many thought that, due to the relative dearth of patients with MDR-TB in resource-rich settings (the traditional sites for randomized trials), clinical trials would be extremely difficult to carry out.

## How to Resolve the Impasse

Recent developments in MDR-TB epidemiology, treatment programs, clinical trials methodology, and TB drug development suggest an end to the present impasse. First, the magnitude of the MDR-TB problem is now understood to be much greater than previously thought. Half a million new cases and an additional 1–1.5 million prevalent cases of MDR-TB were estimated to have occurred globally in 2004 [23]. We also now know that fitness costs are not an inevitable consequence of mutations that confer resistance [24]. And models have illustrated the potential epidemiologic importance of even a few highly fit, drug-resistant strains of M. tuberculosis [25]. There is growing consensus that universal access to high-quality treatment for all patients with TB, including those infected with drug-resistant isolates, is the right of every patient and is sound public health practice [20,26].

Second, MDR-TB treatment programs have expanded dramatically: 40 programs in resource-limited settings are managing treatment for nearly 30,000 patients. These programs receive quality-assured second-line drugs through a pooled-procurement mechanism supported by the Green Light Committee for Access to Second-line Antituberculosis Drugs (see http://www.who.int/tb/dots/dotsplus/management_old/en/index.html) and the Global Fund to fight AIDS, Tuberculosis and Malaria. The requirements for approval by the Green Light Committee have markedly raised standards for MDR-TB treatment in resource-constrained settings. Some of these treatment programs have been operating for nearly 10 years and have developed sophisticated means of delivering supervised ambulatory treatment, guided by drug susceptibility testing—or drug resistance surveys—and other patient-specific characteristics. Although few of these sites have participated in clinical trials, their current standard of care could be raised to that certifiable as “good clinical practice” with limited additional investment.

Third, a clinical trial design has been developed that allows individualization of regimens while evaluating rigorously the safety and activity of a new drug. According to this design, implemented in trials of treatment of drug-resistant HIV infection, patients receive regimens tailored to drug-susceptibility test results and individual characteristics. Patients are randomized to receive either the new drug in addition to the optimized background regimen, or the optimized background regimen alone. As long as randomization is successful in distributing key potential confounding factors equally between study arms, this methodology allows inclusion of patients heterogeneous in many characteristics: prior drug exposure, drug resistance profile, geography, ethnicity, and disease stage. A similar comparative trial design has been used successfully for the pivotal trials showing superiority of the last four antiretroviral drugs approved in the United States (enfuvirtide, tipranavir, darunavir, and maraviroc) [27,28].

Fourth, there are several new classes of antituberculosis drugs in early clinical trials and more in preclinical development [29]. Several agents, which have been used off-label for highly drug-resistant TB in countries with established market economies, could improve MDR-TB treatment efficacy in resource-constrained settings as well [30–32]. The new drug classes have new mechanisms of action. Some also have a narrow spectrum of activity, specific only to M. tuberculosis. Therefore, their activity is not limited by the presence of resistance to currently available drugs and resistance is unlikely to develop through use for other indications. Consequently, they hold great promise for MDR-TB treatment.

## What Is Needed to Move Forward?

Four elements are needed to make MDR-TB treatment trials a reality: money; additional work on the drug pipeline; rigorous, interdisciplinary preclinical work on individual agents and regimens; and an understanding that TB clinical trials need not be a zero–sum endeavor (Box 2).

Despite the frequency of MDR-TB, it is doubtful that a new drug for MDR-TB treatment can repay the cost of its development. Therefore, resources from public institutions, foundations, and public–private partnerships are required to fund clinical trials for MDR-TB. The lack of resources for MDR-TB clinical trials is a reflection of the larger deficit in the field of TB research. Recent estimates indicate that research funding must increase 5-fold in order to meet the targets set by the Global Plan to Stop TB [33].

The new drugs currently in development display promising activity, but a substantial attrition rate is predicted [34]. Therefore funding for basic science, preclinical development, and related research should be increased. Moreover, this work must be integrated with simultaneous efforts to implement clinical trials of currently available drugs. Since clinical trials are expensive, it is critical that new drugs and combinations for MDR-TB be fully evaluated in a preclinical predictor of their efficacy in humans—the animal model of TB treatment. Despite differences in pharmacokinetics and histopathology from human tuberculosis, the mouse model can help to identify promising doses, dosing frequencies, and drug combinations for further evaluation in human trials. Closer collaboration among other, related disciplines (such as medicinal chemistry, microbiology, statistics, and epidemiology) will further enhance drug development efforts.

**Box 2: The Way Forward**

The following elements are essential to ensure the development of optimized regimens, which can be widely implemented, for the treatment of MDR-TB.

- **Increased funding:** 30% of the overall estimated 2005 global TB research allocation, or $120 million, was devoted to drug development [33]. The Global Plan to Stop TB, which was published prior to the alarming reports of XDR-TB, estimated a $4.2 billion gap in drug development resources over 10 years [20]. A recent addendum to the Global Plan, the Global MDR-TB and XDR-TB Response Plan estimates a staggering two-year gap of US$334 million for R&D related to MDR-TB and XDR-TB [37].
- **Additional work on the drug pipeline:** A substantial portion of drug development resources is devoted to human trials of compounds well into the development process. These trials have focused mostly on shortening first-line therapy. Although this is a critical goal, with expected benefits in reducing the incidence of MDR-TB and XDR-TB, its achievement will have no impact on the treatment of prevalent cases. The likelihood that even one of the new compounds currently under development will reach the market by 2010 has been estimated at less than 5% [34]. Efforts in basic science, lead-compound optimization, and early drug discovery will have to be intensified radically to broaden the range of drug targets and to increase the probability and efficiency of new agents reaching the market. Estimated at a paltry $68 million in 2005 [33], additional investment in basic research and drug discovery is essential to ensure that new drugs—and regimens—become a reality.
- **Integration of efforts:** A successful MDR-TB treatment trials approach requires close collaboration along a broad spectrum of disciplines, from basic research to clinical trials. Close interactions between medicinal chemists and microbiologists can accelerate the optimization of an initial compound having promising activity. Similarly, better integration of animal research and human trials will likely result in more efficient development; it is essential to resolve in animals the questions that are most appropriate for that model and to reserve questions that can be answered only in humans for the more expensive, longer clinical trials. In each of these models, it will also be important to integrate testing of new compounds with existing agents used for treatment of resistant TB. Results from these efforts will inform design of parallel trials of new agents in drug-susceptible and drug-resistant TB. Lastly, clinical research must incorporate efforts to identify and validate surrogate endpoints or interim indicators, which will have implications for sample size, duration, and cost of human trials.
- **An understanding that MDR-TB clinical trials need not be a zero-sum endeavor:** Optimization of MDR-TB treatment is essential to fulfill the promise of universal access to effective treatment for all patients with TB. The HIV model has shown that substantial benefits may accrue to acceleration of regulatory approval of antituberculosis agents through development of a parallel track of testing antituberculosis agents in patients with resistant disease. An aggressive MDR-TB clinical trials agenda can also enhance overall support for TB control.

The final element necessary for clinical trials of MDR-TB treatment is acceptance of the importance of a parallel track to evaluate new drugs for drug-resistant TB. The focus of recent and planned TB clinical trials has been the identification of shorter regimens for drug-susceptible TB. The benefit of such regimens would accrue to tens of millions sick with TB, by both curing extant disease and averting acquisition of additional resistance. Consequently, concerns have been raised that evaluating new drugs for MDR-TB may divert scarce resources from the development of shorter regimens for drug-susceptible TB.

This concern echoes similar apprehensions that treating MDR-TB in resource-poor settings would divert the limited resources of national TB control programs and thereby detract from TB control [35]. But just as MDR-TB treatment in resource-limited settings can boost TB control [36], clinical trials of new drugs for MDR-TB treatment can enhance and accelerate drug development efforts.

The threats of MDR- and XDR-TB, moreover, may prove to be a key element in mobilizing increased funding for *all* TB drug development; their prevalence will undoubtedly grow if improved therapy is not made widely available. And the financial and human costs will be even higher. Furthermore, the activity of a new drug may be more easily observed in the context of the relatively weak companion drugs used in MDR-TB treatment than in the presence of other potent drugs used for drug-susceptible disease. Differences in treatment response, which are more easily detected in MDR-TB therapy, would allow smaller and shorter clinical trials. As in HIV, clinical trials in patients with drug-resistant disease may provide a quicker and less expensive path to licensure than demonstrating that a new drug can substantially improve the treatment for drug-susceptible disease.

## The Time Is Now

The devastating outbreak of XDR-TB in KwaZulu-Natal [4] is a reminder of the threat MDR-TB represents to individuals and to global public health. A continued failure to make appropriate therapy for MDR/XDR-TB widely available is unacceptable, as it will have a profoundly negative impact on global TB control.

In recent years, the landscape of TB control has changed dramatically. It is now possible to employ a bold, integrated approach in responding to such outbreaks and averting their spread. The time is right for clinical trials of MDR-TB therapy: existing treatment programs throughout the world can form the backbone for a clinical trials effort; new drug classes are entering clinical trials; and we now have a flexible trial design that allows individualization of therapy while preserving a rigorous randomized evaluation of the safety and efficacy of a new agent. New, optimized regimens that will be identified in clinical trials can be quickly integrated into routine program practice through the expanding global network of MDR-TB treatment programs. Signaling the changing times, at least two pharmaceutical companies, Tibotec and Otsuka, have publicly announced plans to evaluate a new drug in patients with MDR-TB. We have an ambitious goal and responsibility—to successfully treat nearly 1.6 million MDR-TB patients by 2015—and clinical trials to identify safer and more effective treatment are a key to fulfilling that goal. The time for action is now.

## Supporting Information

Alternative Language Abstract S1 Translation of article summary into French by CDM (20 KB DOC).

## Abbreviations

MDR-TB: multidrug-resistant tuberculosis
TB: tuberculosis
XDR-TB: extensively drug-resistant tuberculosis
