# Supplementary material for: Randomized Trials to Optimize Treatment of Multidrug-Resistant Tuberculosis: The time for action is now
Source: PLoS Med. 2007 Nov 6;4(11):e292. doi: 10.1371/journal.pmed.0040292 (PMC2062482; doi:10.1371/journal.pmed.0040292)
Supplement: Supplementary file 1 [file pmed.0040292.sd001.doc]

**French Abstract Carole Mitnick, etal**

La préoccupation de la tuberculose due aux bacilles multirésistants augmente. Le traitement standard, qui est long et mal supporté, échoue souvent. Alors que les essais cliniques randomisés ont réussi à améliorer et abréger la durée du traitement pour la tuberculose due aux bacilles sensibles, ils n’ont jamais été réalisés pour la MDR-TB. Contrairement à ce que l’on a longtemps pensé, ceux-ci sont réalisables et c’est maintenant le moment de les effectuer : le problème des MDR/XDR-TB étant préoccupant, les résultats thérapeutiques étant insuffisants démontrent que la recherche doit se poursuivre. L’expansion des programmes nationaux offre un cadre idéal pour mettre en œuvre les essais cliniques ; les essais cliniques de conception innovatrice permettent une évaluation rigoureuse d’un produit à l’essai chez des sujets traités avec des régimes individualisés. Pour la première fois depuis 30 ans, plusieurs classes de médicaments prometteurs pour lutter contre la MDR-TB sont en cours de mise au point. Ces nouveaux agents doivent être évalués parallèlement dans les infections à bacilles résistants et à bacilles sensibles. La prise de conscience du problème des MDR/XDR-TB peut aider à mobiliser des fonds pour le développement des antituberculeux, de plus, les essais cliniques sur les MDR-TB permettraient d’accélérer l’approbation officiel des nouveaux médicaments.
